# Deciphering complex reticulate evolution of Asian *Buddleja* (Scrophulariaceae): insights into the taxonomy and speciation of polyploid taxa in the Sino-Himalayan region

**DOI:** 10.1093/aob/mcad022

**Published:** 2023-02-01

**Authors:** Fengmao Yang, Jia Ge, Yongjie Guo, Richard Olmstead, Weibang Sun

**Affiliations:** Key Laboratory for Plant Diversity and Biogeography of East Asia, Kunming Institute of Botany, Chinese Academy of Sciences, Kunming 650201, Yunnan, China; Yunnan Key Laboratory for Integrative Conservation of Plant Species with Extremely Small Populations, Kunming Institute of Botany, Chinese Academy of Sciences (CAS), Kunming 650201, Yunnan, China; Key Laboratory for Plant Diversity and Biogeography of East Asia, Kunming Institute of Botany, Chinese Academy of Sciences, Kunming 650201, Yunnan, China; Yunnan Key Laboratory for Integrative Conservation of Plant Species with Extremely Small Populations, Kunming Institute of Botany, Chinese Academy of Sciences (CAS), Kunming 650201, Yunnan, China; Germplasm Bank of Wild Species of China, Kunming Institute of Botany, Chinese Academy of Sciences, Kunming 650201, Yunnan, China; University of Chinese Academy of Sciences, Beijing 100049, China; Department of Biology and Burke Museum, University of Washington, Seattle, WA 98195, USA; Key Laboratory for Plant Diversity and Biogeography of East Asia, Kunming Institute of Botany, Chinese Academy of Sciences, Kunming 650201, Yunnan, China; Yunnan Key Laboratory for Integrative Conservation of Plant Species with Extremely Small Populations, Kunming Institute of Botany, Chinese Academy of Sciences (CAS), Kunming 650201, Yunnan, China

**Keywords:** *Buddleja*, phylogenomics, reticulate evolution, polyploidy, plastid genome, low-copy nuclear gene

## Abstract

**Background and Aims:**

Species of the genus *Buddleja* in Asia are mainly distributed in the Sino-Himalayan region and form a challenging taxonomic group, with extensive hybridization and polyploidization. A phylogenetic approach to unravelling the history of reticulation in this lineage will deepen our understanding of the speciation in biodiversity hotspots.

**Methods:**

For this study, we obtained 80 accessions representing all the species in the Asian *Buddleja* clade, and the ploidy level of each taxon was determined by flow cytometry analyses. Whole plastid genomes, nuclear ribosomal DNA, single nucleotide polymorphisms and a large number of low-copy nuclear genes assembled from genome skimming data were used to investigate the reticulate evolutionary history of Asian *Buddleja*. Complex cytonuclear conflicts were detected through a comparison of plastid and species trees. Gene tree incongruence was also analysed to detect any reticulate events in the history of this lineage.

**Key Results:**

Six hybridization events were detected, which are able to explain the cytonuclear conflict in Asian *Buddleja*. Furthermore, PhyloNet analysis combining species ploidy data indicated several allopolyploid speciation events. A strongly supported species tree inferred from a large number of low-copy nuclear genes not only corrected some earlier misinterpretations, but also indicated that there are many Asian *Buddleja* species that have been lumped mistakenly. Divergent time estimation shows two periods of rapid diversification (8–10 and 0–3 Mya) in the Asian *Buddleja* clade, which might coincide with the final uplift of the Hengduan Mountains and Quaternary climate fluctuations, respectively.

**Conclusions:**

This study presents a well-supported phylogenetic backbone for the Asian *Buddleja* species, elucidates their complex and reticulate evolutionary history and suggests that tectonic activity, climate fluctuations, polyploidization and hybridization together promoted the diversification of this lineage.

## INTRODUCTION

Reticulation in evolution can occur as a result of hybridization, introgression or lateral gene transfer (Mallet *et al.*, 2016; Suvorov *et al.*, 2022) and is believed to be one of the main driving forces in the diversification of angiosperms (Mallet *et al.*, 2016; Debray *et al.*, 2022; Suvorov *et al.*, 2022). Hybridization often occurs in lineages that have undergone recent radiations when their habitats undergo dramatic change, such as during climatic fluctuations and anthropogenic disturbance (Rieseberg and Willis, 2007; Abbott *et al.*, 2013; Estep *et al.*, 2014). Allopolyploids arise from the integration of distinct parental chromosome sets (Van de Peer *et al.*, 2017), which will lead to a highly dynamic genome (Pontes *et al.*, 2004; Zhou *et al.*, 2011) and might help plants to survive and thrive in precarious environmental conditions (Estep *et al.*, 2014; Soltis *et al.*, 2014; Edgeloe *et al.*, 2022). Hybridization can also result in adaptive introgression, allowing species to adapt to new environments (Owens *et al.*, 2016; Ma *et al.*, 2019; Oziolor *et al.*, 2019). Given that 25 % of all plant species are thought to have been involved in interspecific hybridization (Mallet, 2005), the construction of phylogenetic networks is particularly important for understanding the evolutionary history of plant species, especially that of recently radiated taxa (Mallet *et al.*, 2016; Goulet *et al.*, 2017).

Polyploidy (either allopolyploidy or autopolyploidy) is prevalent in angiosperms (Van de Peer *et al.*, 2017). Polyploids, or plants that have undergone whole-genome duplications (WGDs), were once considered to be ‘evolutionary dead ends’ or ‘evolutionary noise’, because WGDs were thought to have only limited long-term evolutionary potential (Stebbins, 1950). Indeed, one study based on phylogenetic approaches has shown that polyploids have higher extinction rates and lower speciation rates than their diploid relatives (Mayrose *et al.*, 2011). However, many recent studies have demonstrated that polyploidy is positively correlated with species adaptation and diversification (Levin and Soltis, 2018; Ren *et al.*, 2018; Han *et al.*, 2020), and WGD is now recognized as a major evolutionary force in plants (Soltis *et al.*, 2014; Van de Peer *et al.*, 2017; Wu *et al.*, 2020).

Phylogenetic study of neopolyploids has proved to be challenging (Rothfels, 2021), because many polyploids arise from hybridization (allopolyploids; Funk and Omland, 2003; Rieseberg and Willis, 2007; Barker *et al.*, 2016). An allopolyploid typically exhibits reproductive isolation from its parents, and allopolyploidy is generally considered to be a common mode of speciation (Ramsey and Schemske, 2002; Rieseberg and Willis, 2007; Abbott *et al.*, 2013). Despite advances in the use of genomic data to resolve reticulate evolution in allopolyploid species (Guo *et al.*, 2019; Jia *et al.*, 2022), building a comprehensive evolutionary history for large taxonomic groups remains difficult (Diaz-Perez *et al.*, 2018; Rothfels, 2021; Debray *et al.*, 2022; Suissa *et al.*, 2022).

**Fig. 1.**
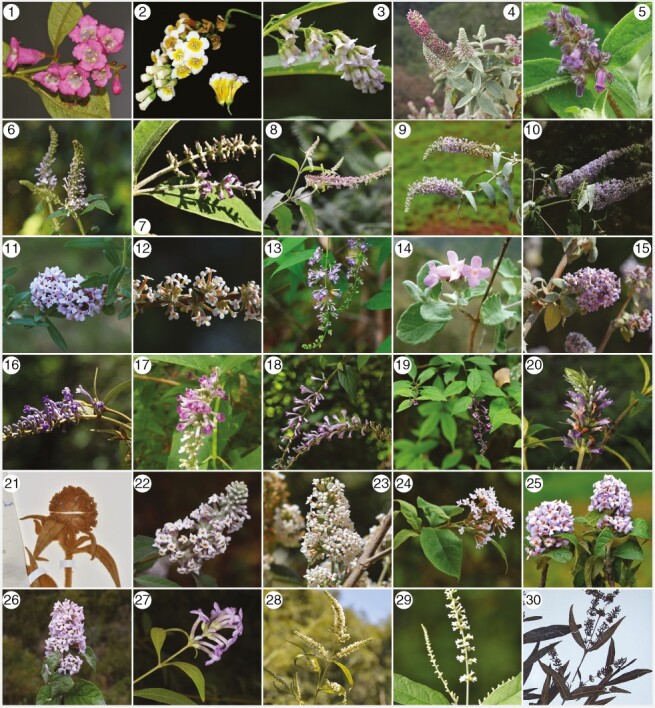
Photographs of Asian *Buddleja* taxa: (1) *B. colvilei*; (2) *B. sessilifolia*; (3) *B. forrestii*; (4) *B. macrostachya*; (5) *B. nivea*; (6) *B. myriantha*; (7) *B. candida*; (8) *B. albiflora*; (9) *B. fallowiana*; (10) *B. davidii*; (11) *B. alternifolia*; (12) *B. tsetangensis*; (13) *B. jinsixiaensis*; (14) *B. caryopteridifolia*; (15) *B. crispa*; (16) *B. curviflora*; (17) *B. japonica*; (18) *B. lindleyana*; (19) *B. lindleyana* (GJ68); (20) *B. yunnanensis*; (21) *B. subcapitata*; (22) *B. officinalis*; (23) *B. paniculate*; (24) *B. delavayi*; (25) *B. microstachya*; (26) *B.* sp. 1; (27) *B. brachystachya*; (28) *B. asiatica*; (29) *B*. *asiatica* = *B. subserrata*; and (30) *B. bhutanica*.

*Buddleja* L. (Scrophulariaceae) are typically shrubs or small trees (Norman, 2000). Plants in this genus are known as butterfly bushes owing to their attractiveness to butterflies (Stuart, 2006) and are widely cultivated and important components in horticulture and human culture (Fig. 1; Tallent-Halsell and Watt, 2009). Some species (e.g. *Buddleja davidii*; Tallent-Halsell and Watt, 2009) have escaped cultivation and have become problematic and invasive in natural areas. In China, the genus is known as ‘Zui Yu Cao’, and the leaves of certain species (e.g. *B. lindleyana* and *B. curviflora*; Houghton, 1984) are used in fishing owing to their toxicity to fish. Some species have culinary applications and are used as medicines (e.g. *B. officinalis*, *B. asiatica*, *B. davidii* and *B. lindleyana*; Houghton, 1984; Li *et al.*, 2020; Yan XX *et al.*, 2021).

The genus *Buddleja* comprises ~90 species in the tropical, subtropical and warm-temperate areas of Africa, Asia and North and South America (Norman, 2000; Chau *et al.*, 2017). The Asian *Buddleja* clade is well supported as being monophyletic (Chau *et al.*, 2017). In descriptive taxonomy, this is a notoriously difficult group of species, which is reflected in the frequent changes to species delimitation in the group (Marquand, 1930; Leeuwenberg, 1979; Li, 1982, 1988; Bao, 1983; Zhang *et al.*, 2014; Ge *et al.*, 2018) and controversial taxonomic systems (Bentham, 1846; Marquand, 1930; Leeuwenberg, 1979; Li, 1982; Li and Leeuwenberg, 1996; Norman, 2000; Oxelman, 2004; Chau *et al*., 2017). The Flora of China, in addition to several other studies, currently list 27 species in the Asian *Buddleja* clade (Li and Leeuwenberg, 1996; Norman, 2000; Liu and Peng, 2004, 2006; Zhang *et al.*, 2014; Zhu *et al.*, 2014; Ge *et al.*, 2018). The Sino-Himalayan region of Southeast Asia is the centre of diversity for Asian *Buddleja*, harbouring 25 of the 27 Asian *Buddleja* species (all except for *B. curviflora* and *B. japonica*; Wu *et al.*, 2010). The tectonic activity and climate fluctuations that took place in the Sino-Himalayan region during the Miocene are believed to have played a crucial role in the diversification of plant species in this region (Ding *et al.*, 2020). However, whether the diversification of the Asian *Buddleja* is related to those palaeoclimatic and geological events has not yet been investigated.

Asian *Buddleja* species show a high proportion of polyploid species, and different ploidy levels are observed, including diploids, tetraploids, hexaploids, dodecaploids, 16-ploids and 24-ploids (2*n* = 38, 76, 114, 228, 300 and 456; Chen *et al.*, 2007). Polyploidy might facilitate the adaptation of *Buddleja* to an alpine environment and promote niche diversification and speciation in the genus in the Sino-Himalayan region (Chen *et al.*, 2007).

Interspecies hybridization is common in *Buddleja*, owing to overlaps in distribution, flowering period and pollinators between species (Liao *et al.*, 2021). Twenty-five natural hybrids of *Buddleja* have been inferred based on morphological characteristics, 19 from the Neotropics and six from the Old World taxa (Norman, 2000). Two natural Asian *Buddleja* hybrids have been confirmed with both morphological and molecular evidence (Liao *et al.*, 2015, 2021). It is thought that hybridization might promote speciation via allopolyploid speciation or via ‘adaptive introgression’ allowing the plants to adapt to new ecological niches (Abbott *et al.*, 2013). Given that polyploidy, hybridization and cytonuclear conflicts are common in Asian *Buddleja* (Chen *et al.*, 2007; Chau *et al.*, 2017), events leading to reticulation might play an important role in the diversification of this lineage. Morphological continuity, low sequence differentiation and hybridization or polyploidization between the newly diverged lineages can exacerbate the difficulties facing taxonomic and polygenetic research (Stoughton *et al.*, 2018). Previous studies, although revealing the phylogenetic relationships between *Buddleja* species worldwide, failed to cover all Asian species and did not explain the observed cytonuclear conflicts (Chau *et al.*, 2017, 2018). More informative molecular sequences and extensive sampling are urgently needed to illustrate the phylogenetic structure and complex reticulate evolutionary history in this lineage.

We used a large number of low-copy nuclear (LCN) genes, single nucleotide polymorphisms (SNPs), nuclear ribosomal DNA (nrDNA) sequences and whole plastid genomes assembled from data generated by genome skimming technology to illustrate the phylogenetic relationships and evolutionary history of Asian *Buddleja* species. The reticulate relationships in this lineage were highlighted initially because of cytonuclear conflicts and were confirmed with gene tree incongruence and Bayesian clustering. The aims of the present study were as follows: (1) to reconstruct a robust phylogenetic backbone for the Asian *Buddleja* clade and lay the foundations for future species delimitation in this lineage; (2) to explore the reticulate evolutionary history of Asian *Buddleja*; and (3) to infer the evolutionary history of the Asian *Buddleja* lineage and its potential associations with tectonic activity and climatic fluctuations.

## MATERIALS AND METHODS

### Taxon sampling, DNA extraction and sequencing

A total of 80 accessions (Supplementary data Table S1), including data from 64 newly sequenced accessions and 16 sequences already available from GenBank, were included in this study. Our samples represented 32 taxa, including 27 species, three hybrids and two undescribed species. All voucher specimens are listed in the Supplementary data (Table S1).

Total DNA was extracted from silica gel-dried leaf tissues using a cetyltrimethylammonium bromide (CTAB) method. Purified DNA was fragmented, and short insert (500 bp) libraries were constructed according to the manufacturer’s instructions on an Illumina HiSeq X Ten platform, and were then sequenced on an Illumina HiSeq platform with a read length of 300–500 bp, by a commercial service (Beijing Ori-Gene Science and Technology).

### Flow cytometry

Flow cytometry analyses were carried out at the Laboratory of Molecular Biology of Germplasm Bank of Wild Species in Southwest China following the protocol described by Doležel *et al.* (2007). Thirty-three leaf samples of 29 *Buddleja* taxa were collected (one or two samples of each of the 26 species and one sample of each of the three hybrids; Supplementary data Table S2). About 0.5 cm^2^ of fresh young leaf tissue was chopped with a razor blade in a Petri dish containing 0.8 mL ice-cold MGb buffer. The resulting solutions were subsequently filtered through 40 µm nylon mesh to obtain the cell nuclei; 50 µL of propidium iodide solution (1 mg/mL) and 5.0 µL of RNAse (100 µg/mL) were added to each sample, and the samples were then stored in the dark for 0.5–1 h. The nuclear DNA content was measured on a flow cytometer using the DNA 2C-values of *Zea mays* L. and *Solanum lycopersicum* L. as the internal standards. The number of nuclei was normalized to 10 000 per sample using the fluorescently labelled propidium iodide in each experiment, the cross-validation (CV) % was controlled to within 5 %, and the nuclei were surveyed by BD FACSCalibur. The relative nuclear DNA content of each plant sample was then determined by comparison with the peak positions of the nuclei from the internal standards. The ploidy level was determined based on the ratio of G1 peak positions of the diploid *B. asiatica* and tetraploid *B. davidii* nuclei.

### Sequence assembly, annotation and alignment

The paired-end reads were filtered using fastp v.0.20.1 (Chen *et al.*, 2018) with the default parameters. The plastid genomes were assembled using the GetOrganelle pipeline v.1.7.1 (Jin *et al.*, 2020) with the recommended parameters for embryophyte plant plastome assembly (https://github.com/Kinggerm/GetOrganelle). Annotation of plastids was performed using the plastid genome annotator (PGA; Qu *et al.*, 2019), and the recommended *Amborella trichopoda* plastome genome was selected as a reference. The results were aligned with five published plastid genomes from Asian *Buddleja* species (Ge *et al.*, 2018) using MAFFT v.7.3.08 (Katoh and Standley, 2013), and the annotations were checked manually in Geneious v.9.0.2 (Biomatters, Auckland, New Zealand). The coding sequences (CDS) regions were extracted from each plastid using Geneious and aligned using MAFFT. The nrDNA sequences were assembled using GetOrganelle with the recommended parameters for plant nuclear ribosomal RNA assembly (https://github.com/Kinggerm/GetOrganelle). The assembled nrDNA sequences were aligned and checked manually in Geneious. Aligned whole plastid sequences and CDS regions were trimmed using Gblocks (Talavera and Castresana, 2007) in PhyloSuite (Zhang *et al.*, 2020) with the default parameters. However, owing to the uneven quality of the nrDNA assembly, aligned nrDNA sequences were trimmed using Gblocks with half gape position allowed (-b5 = h).

Phylogenetic analyses were implemented using whole plastid sequences and nrDNA sequences using RAxML v.8.2.12 with 1000 bootstraps, and with the ‘GAMMAI’ substitution model, as indicated by Abadi *et al.* (2019).

### LCN gene construction and discovery of nuclear variation

Given that there is only one complete published genome within the genus *Buddleja* to date, the LCN genes were identified following the methods described by Ma *et al.* (2021). The protein-coding genes of *Buddleja alternifolia* (Ma *et al.*, 2021) and *Tectona grandis* (a woody member of the Lamiaceae; Zhao *et al.*, 2019) were analysed with Orthofinder (Emms and Kelly, 2015) to identify the orthologous gene clusters. The HybPiper pipeline v.1.3.1 (Johnson *et al.*, 2016) was used with the default settings for targeting genes. Gene sequences were aligned using MAFFT and converted to codon alignments using pal2nal (Suyama *et al.*, 2006). Aligned codons were trimmed using trimAl (Capella-Gutiérrez *et al.*, 2009). The gene trees were constructed using IQtree v.1.6.12 (Nguyen *et al.*, 2014) with 1000 bootstrap replicates. Species trees were inferred using ASTRAL-III v.5.7.1 (Zhang *et al.*, 2018) based on multiple gene trees. Conflicts between plastid, nrDNA and species trees were examined using phytools (Revell, 2012) in R.

We used BWA v.07.17 (Li and Durbin, 2009) to make an index for the genome of *B. alternifolia* and used BWA-MEM with the default parameters to map the filtered reads to the reference genome. Variant detection was carried out using the genome analysis toolkit GATK4 (McKenna *et al.*, 2010) following the best practices workflow for variant discovery (DePristo *et al.*, 2011). Hard filters were implemented on the raw SNP dataset with the following filter parameters; (1) SNPs with read depth >200 or <5; (2) SNPs with missing rate of >80 %; (3) MAF >0.05; and (4) non-biallelic SNPs.

### Hybrid analysis

To reduce the computational burden and to increase the accuracy of speculation, 32 samples with relatively high sequencing quality and low missing sequence rate (Supplementary data Table S2) were chosen to form a sub-dataset. *Buddleja caryopteridifolia*, *B. myriantha* and *B. jinsixiaensis* were excluded, owing to the very high rate of missing sequences in the gene matrix. Finally, 23 species and a hybrid plant were selected to simulate the reticulate evolutionary history of Asian *Buddleja*. PhyloNet (Than *et al.*, 2008) was used to infer possible hybrid events with the InferNetwork_MPL geneTreeList function and the parameters ‘-x 6 -b 50’. The optimal number of hybridization events was estimated by searching the global optimum of the likelihood (Cao *et al.*, 2019). The optimum phylogenetic networks were visualized in Dendroscope (Huson *et al.*, 2007). A Bayesian clustering analysis was also performed using Admixture (Alexander *et al.*, 2009) with the same samples as those used in the PhyloNet analysis. We tested numbers of clusters from two to seven, with the optimal number of clusters estimated via the lowest cross-validation error rate. We used the package ‘Pophelper’ (Francis, 2017) in R v.3.6.3 (R Core Team, 2018) to visualize the Admixture results.

### Molecular dating

Given that the homogeneity of chloroplast sequences is much higher than that of LCN genes and that multiple published chloroplast genomes could provide more options for a calibration point, the concatenated plastid CDS regions from the non-redundant dataset were used to estimate the divergence time of Asian *Buddleja*. Divergence time was estimated in BEAST v.1.10 (Drummond and Rambaut, 2007). Two calibration points were chosen from TimeTree (http://timetree.org/). The root of the time tree was constrained to 71 Mya, with a normal distribution and s.d. of 10 Mya. The ancestral node of the Scrophulariaceae samples selected in this study was constrained to 44 Mya, with a normal distribution and s.d. of 10 Mya. The BEAST analyses were performed using an uncorrelated log normal relaxed clock with a Yule tree prior, a random starting tree and ‘Gamma + Invariant Sites’ as the model of sequence evolution. The Markov chain Monte Carlo (MCMC) analysis was run for 200 million generations, sampling every 1000 generations, and the first 20 million samples were discarded as burn-in. Convergence of the MCMC runs was checked using Tracer v.1.6. Tree Annotator v.1.8.0 (Drummond *et al.*, 2012) was used to summarize the set of post-burn-in trees and their parameters to produce a maximum clade credibility chronogram showing the mean divergence time estimates with 95 % highest posterior density (HPD) intervals. Figtree v.1.4.4 (http://tree.bio.ed.ac.uk/software/figtree/) was used for image drawing of the time tree. Lineage-through-time (LTT) plots were drawn using the APE package (Paradis *et al.*, 2004) in R.

## RESULTS

### Determination of ploidy diversity using flow cytometry

The levels of ploidy of the 33 samples were determined. Fifteen samples representing *B. alternifolia*, *B. asiatica* (GJ1 & GJ34), *B. caryopteridifolia*, *B. crispa*, *B. curviflora*, *B. jinsixiaensis*, *B. lindleyana* (GJ5 & GJ68), *B. officinalis*, *B. paniculata*, *B. tsetangensis*, *B. yunnanensis*, *B. crispa* × *B. paniculata* and *B.* × *wardii* were considered to be diploids. The rest were presumed to be polyploids, including seven tetraploids (*B. brachystachya*, *B. candida*, *B. davidii*, *B. fallowiana*, *B. macrostachya*, *B. myriantha* and *B. sessilifolia*), five hexaploids (*B. albiflora*, *B. delavayi*, *B. forrestii*, *B.* sp. 1 and *B.* sp. 1 × *delavayi*), one 12-ploid (*B. nivea*) and one 24-ploid (*B. colvilei*); the *B. microstachya* samples were found to consist of both tetraploid and hexaploid samples, and the *B. macrostachya* samples were found to consist of both hexaploid and 12-ploid samples. The ploidy levels of eight Asian *Buddleja* species and hybrids were determined using flow cytometry and are reported here for the first time. Tetraploid is a new ploidy level for *B. brachystachya*, and the ploidy levels determined for the remaining species are consistent with those published previously. The available cytological data are shown in the Supplementary data (Table S2).

### Nuclear and plastid gene assembly and SNP calling

The number of clean reads for genome skimming data ranged from 6.6 million (*B. myriantha* GJ37) to 38.7 million (*B. yunnanensis* GJ75) with an average of 18.6 million (Supplementary data Table S3). In order to prevent the bias caused by the uneven sample depth in LCN gene assembly, ten resequenced samples downloaded from GenBank were reduced to 20.0 million reads.

A total of 10 791 LCN genes were discovered using OrthoFinder. The number of genes recovered for each sample varied from 9240 (*B. jinsixiaensis*) to 10 763 (*B. davidii*). After trimming away those with a maximum missing rate >30 %, 10 429 LCN genes were used to construct the ASTAL species tree. The nrDNA sequence of *B. bhutanica* GJ42 was discarded owing to its short and fragmented sequences. The trimmed nrDNA data matrix comprised 8724 characters, of which 1666 were parsimony-informative sites. Consistent with previous research (Ge *et al.*, 2018), the plastomes of *Buddleja* showed typical quadripartite architecture (Supplementary data Fig. S1). The trimmed whole plastome data matrix comprised 158 290 characters, of which 1605 were parsimony-informative sites. After filtering, a total of 87 039 SNPs were obtained from the 32 high-quality samples.

### Phylogenetic reconstruction

**Fig. 2.**
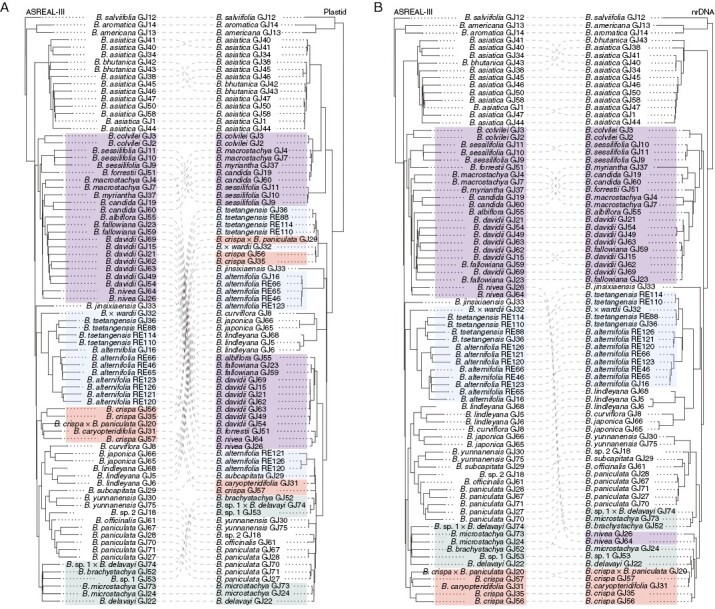
Tanglegram of the ASTRAL-III species tree and (A) plastid tree or (B) nuclear ribosomal DNA (nrDNA) tree. The two ASTRAL species trees are identical topologically, but rotated at some nodes to match up with the plastid or nrDNA trees. Different colour blocks represent clades with obvious cytonuclear conflict.

The phylogenetic structure of the ASTRAL species tree is generally in accordance with that of the nrDNA tree (Fig. 2B). There are, however, complex conflicts between the plastid and species trees (Fig. 2A).

Phylogenetic reconstruction based on the plastid dataset indicated that *Buddleja asiatica*, *B. bhutanica* and five polyploid species (*B. sessilifolia*, *B. colvilei*, *B. macrostachya*, *B. myriantha* and *B. candida*) composed plastid clade 1, and the remaining species composed plastid clade 2 [bootstrap support (BS) = 100 %; Fig. 2A]. The plastid phylogeny suggested that *B. alternifolia* is polyphyletic, because the three *B. alternifolia* samples (RE121, RE123 and RE126) sampled in Sichuan clustered together with *B. subcapitata* and *B. caryopteridifolia*, while the remaining samples formed a clade with *B. jinsixiaensis* (Fig. 2A). In addition, the *B. crispa* complex (*B. crispa* and *B. caryopteridifolia*; Leeuwenberg, 1979) was also revealed to be polyphyletic in the plastid phylogeny, with GJ31 and GJ57 being far apart from the other two samples of *B. crispa* (GJ35 and GJ56).

**Fig. 3.**
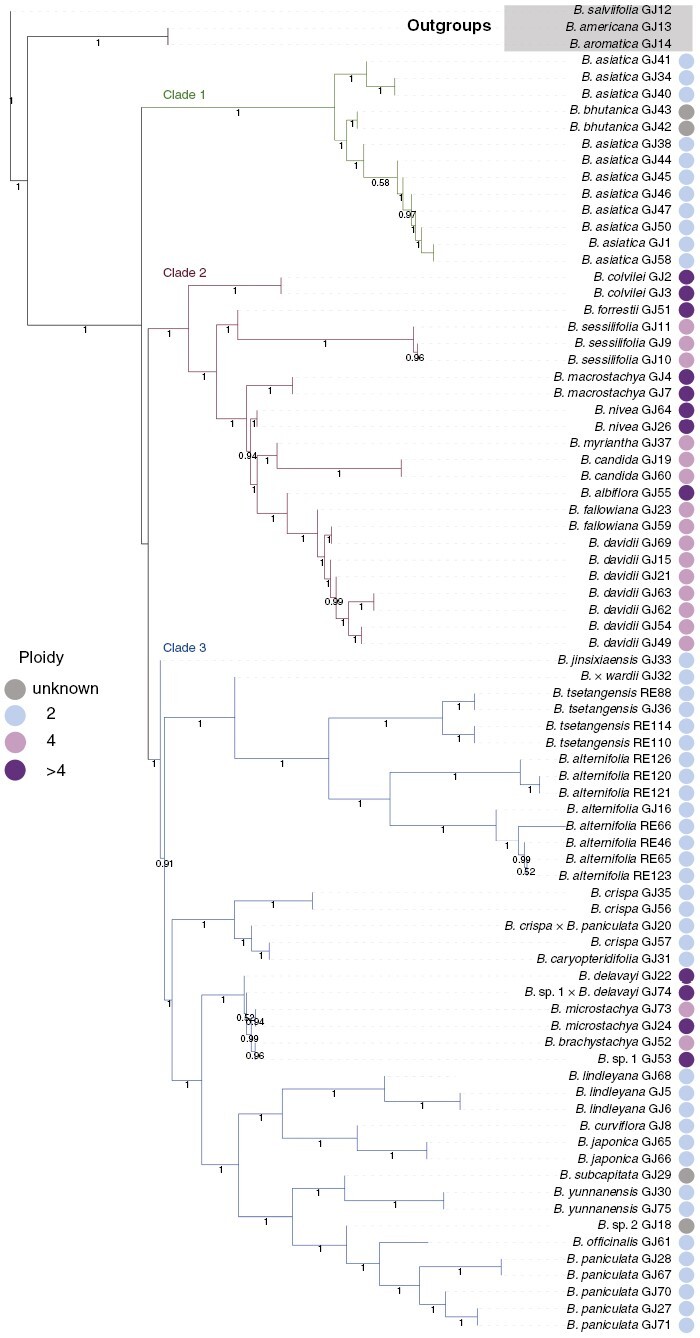
ASTRAL-III species tree. The ploidy of each species is indicated by the coloured circles.

The species tree inferred from the LCN genes strongly (bootstrap support BS = 100 %) supported three clades in Asian *Buddleja*: ASTRAL clade 1 included *B. asiatica* and *B. bhutanica*; ASTRAL clade 2 included ten polyploid species with mainly Himalayan–Hengduan Mountains distribution (Chen *et al.*, 2007; Wu *et al.*, 2010); and ASTRAL clade 3 included the remaining species (Fig. 3). A notable conflict between the plastid tree and the species tree is visible in the cases of the five polyploid species (*B. forrestii*, *B. nivea*, *B. albiflora*, *B. fallowiana* and *B. davidii*), which formed a clade together with another five polyploid species in the species tree, but nested within plastid clade 2 (Fig. 2A). The species tree also supported the monophyly of both the *B. alternifolia* complex and *B. crispa* complex, which did not appear as clades in the plastid tree (Fig. 2A). Although the nrDNA tree shared a similar topography to the species tree, the position of *B. nivea* was in dramatic conflict (Fig. 2B).

### Network and gene flow analysis

**Fig. 4.**
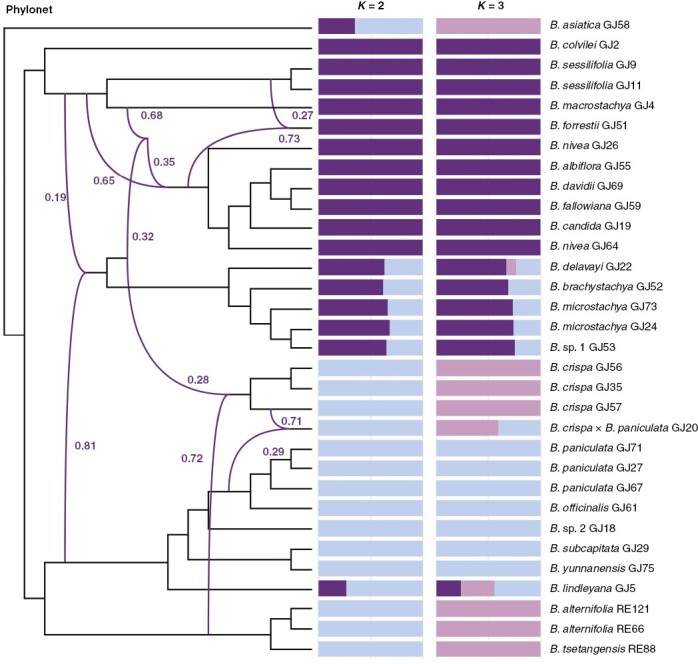
Best-supported species networks inferred with PhyloNet for the 32 samples, and best two scenarios from Bayesian clustering analysis inferred from Admixture with the same samples.

A sub-dataset of 32 samples, including 23 species and one hybrid, was used to process the PhyloNet analysis and the Bayesian clustering (Supplementary data Table S3). Up to six hybridization events among the clades of *Buddleja* were examined in PhyloNet. Six reticulate evolutionary events proved to be the best scenario, based on the global optimum of the likelihood. The best two values of *K* (the number of ancestral populations) in the Bayesian clustering analysis, as indicated by CV error values, were two and three. A reticulation event and the mixture of two genetic backgrounds are clearly visible in *B. paniculata* × *B. crispa* GJ20, confirming its hybrid origin. PhyloNet analysis also suggested three hybridization events (Fig. 4) in five polyploid species (*B. forrestii*, *B. nivea*, *B. albiflora*, *B. fallowiana* and *B. davidii*), in which there were clear conflicts between the plastid and nrDNA trees (Fig. 2A). *Buddleja forrestii* might have originated as a hybrid between *B. sessilifolia* and the ancestor of another four species. In addition, four polyploids (*Buddleja delavayi*, *B. microstachya*, *B. brachystachya*, and *B.* sp. 1) in clade 3 of the species tree contained two reticulation events, and the Bayesian clustering results also supported admixture. The *B. crispa* complex is likely to have received gene flow from the *B. alternifolia* cluster, in addition to a ‘ghost introgression’ (donors of gene flow might be extinct or unsampled).

### Molecular dating

**Fig. 5.**
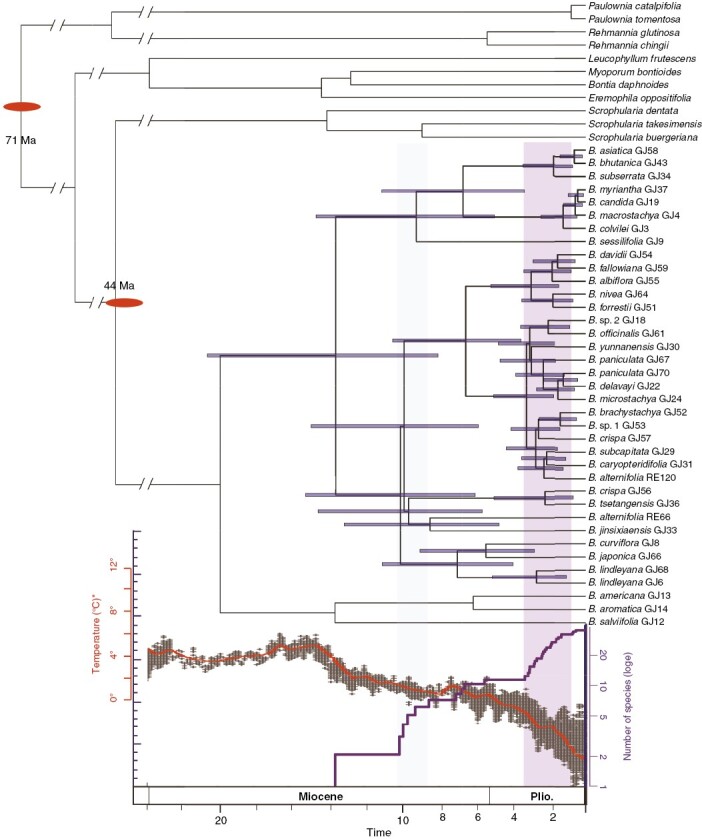
BEAST analysis of divergence times based on plastid data, with trends in global climate change over 30 Mya depicted (red line) and lineage-through-time plot for this taxon (purple line).

Divergence time estimates based on the CDS region of the plastid indicated that the divergence time of the two clusters in the plastid tree was 14.2 Mya (95 % HPD: 8.44–21.57 Mya). The LTT plots suggested that the Asian *Buddleja* clade experienced two rapid diversifications, at 8–10 and 0–3 Mya (Fig. 5). The topography of the plastid tree cannot reflect that of the real species tree, owing to the reticulation; however, this does not affect the estimated dating of diversification in *Buddleja*.

## DISCUSSION

### The performance of the different phylogenetic trees

In this study, we reconstructed the phylogenetic relationships within Asian *Buddleja* using biparental (nrDNA sequences and LCN genes) and maternal (whole plastid) sequences. Phylogenetic structures inferred using these different sets of sequences have unique advantages and potential biases (Álvarez and Wendel, 2003; Nieto Feliner and Rosselló, 2007; Gitzendanner *et al.*, 2018). A comprehensive assessment of all phylogenetic trees should allow us to have a relatively accurate understanding of the evolutionary history of Asian *Buddleja*.

The phylogenetic structure resulting from analysis of the nrDNA data showed the same three-cluster structure as the ASTRAL species tree (Fig. 2B). However, conspicuous differences in the length of the nrDNA sequence assembly, owing to uneven sequencing quality, caused bias in the phylogenetic relationships at the species level, such as in the cases of *B. fallowiana* and *B. microstachya*. In addition, the unexpected position of *B. nivea* might be caused by the short length of the nrDNA assembly of the two samples (GJ26 and GJ64), given that *B. nivea* grouped with many polyploids in both the plastid and species trees (Fig. 2A).

Owing to the lack of genomic resources in the genus *Buddleja*, methods designed to identify orthologous genes were not able to avoid paralogous genes in this lineage (Xiong *et al.*, 2022). The presence of paralogous genes can lead to problems in inference of a species tree (Fitch, 1970; Cheon *et al.*, 2020; Kapli *et al.*, 2020). Recent studies (Smith and Hahn, 2021*a*; Yan Z *et al.*, 2021) have suggested that, in certain circumstances, species tree inference in the presence of paralogues is as accurate as phylogenetic analyses using orthologues. Many approaches can also reduce the adverse effects of paralogous genes on the construction of a species tree, such as using quartet-based gene tree methods (e.g. ASTRAL; Yan Z *et al.*, 2021) and increasing the number of loci used in phylogenetic inference (Smith and Hahn, 2021*b*). In the present study, we used a large number of LCN genes and the coalescence method (ASTRAL-III) to infer the best possible species tree of Asian *Buddleja*.

### *Implications for species delimitation of Asian* Buddleja

Species delimitation of *Buddleja* in Asia is notoriously difficult (Li, 1982) owing to the transitional traits between species (e.g. the *B. crispa* complex) and huge variation within species (e.g. *B. davidii*). Moreover, hybridization creates many individuals with transitional morphology (Liao *et al.*, 2015), which results in conflict among the different classification systems (Leeuwenberg, 1979; Li, 1982). Through extensive sampling and multiple sequence construction, the present study yielded a strong phylogenetic backbone for this lineage, allowing us to provide evidence for the delimitation of certain species.

Three samples of *B. asiatica* (GJ34, GJ40 and GJ41) collected in Nepal and Tibet formed a sister group to the remaining samples of *B. bhutanica* and *B. asiatica*, implying that these specimens exhibit high genetic differentiation from the other *Buddleja* specimens in this clade (Fig. 3). Through morphological comparison (Supplementary data Table S4; Fig. S2) and examination of the original descriptions and type specimens (997787 BM! and 521826 BM!), these samples with Himalayan distribution might refer to *Buddleja subserrata* (Hamilton, 1825), a synonym of *B. asiatica*, and suggests that *B. subserrata* might be recognized as a distinct species.

Based on our phylogenetic reconstructions, the *B. crispa* complex includes at least three species: species 1 includes GJ35 and GJ56 (*B. crispa*); species 2 includes GJ31 (*B. caryopteridifolia*); and species 3 includes GJ57, which is morphologically different from *B. caryopteridifolia* (Supplementary data Table S5; Fig. S3), which suggests that it might be a distinct species. *Buddleja crispa* is widely distributed and is prone to hybridization with other species (Liao *et al.*, 2015, 2021), resulting in morphological continuity. Thus, 15 species and many varieties were reduced to synonyms (Leeuwenberg, 1979). Our study not only confirmed the species position of *B. caryopteridifolia* but also implied that there are synonyms that might have been mistakenly incorporated into *B. crispa*.

*Buddleja officinalis* and *B. paniculata* are considered morphologically similar to each other and are easily confused. *Buddleja paniculata* typically has a white corolla, with the corolla tube being both shorter and thinner than that typical of the lilac *B. officinalis* (specimen numbers 263011 A!, 276688 GZU!, 6968182 BR! and 1096401 K!; Fig. 1; Leeuwenberg, 1979; Li, 1992). Both species are known locally as ‘Mi Meng Hua’ in Chinese (Yang Fengmao, personal observation). The Chinese name ‘mun-chua’ (another common name of ‘Mi Meng Hua’) is mentioned in the original description of *B. officinalis* (Maximowicz, 1880), whereas *B. paniculata* was first introduced as having the Chinese name ‘Hou Yao Zui Yu Cao’ in 1982 (Li, 1982). Flora Yunnanica (Bao, 1983) lists only *B. officinalis*, and most ‘Mi Meng Hua’ plants sampled in Yunnan have been identified as *B. officinalis* (e.g. Liao *et al.*, 2015; Yan XX *et al.*, 2021; Yang *et al.*, 2023). However, molecular and morphological comparisons (Supplementary data Table S6; Fig. S4) suggest to us that the ‘Mi Meng Hua’, widely distributed throughout Yunnan, is in fact *B. paniculata* (‘Hou Yao Zui Yu Cai’ in Chinese).

The *B. lindleyana* sample GJ68 exhibits large morphological and molecular differences from other samples (GJ5 and GJ6) of *B. lindleyana*: it has distinctly serrated leaves [Fig. 1 (19)] and was once treated as a variety *B. lindleyana* var. *sinuatodentata* (Marquand, 1930). Our study reveals that it might be a distinct taxonomic unit that needs further study.

The specimen (0022547 KUN!) of GJ18 was identified as a new species, *Buddleja adenocarpa* B. S. Sun, in 1960, and Leeuwenberg reidentified it as *B. brachystachya*. Our study showed that GJ18 did not cluster with the *B. brachystachya* samples collected around the type locality, and therefore supported it as a distinct species. Careful comparison and further verification should be carried out in the future.

### *Reticulate evolutionary history of Asian* Buddleja

Hybridization in extant species of Asian *Buddleja* has been documented and studied extensively (Leeuwenberg, 1979; Liao *et al.*, 2015, 2021). The complex and deep cytonuclear conflicts revealed in the present study indicated that allopolyploidy, hybridization and introgression might have been present throughout the evolutionary history of Asian *Buddleja*.

Five polyploid species (*B. forrestii*, *B. nivea*, *B. albiflora*, *B. fallowiana* and *B. davidii*) formed a monophyletic group with another five polyploid species in the ASTRAL species tree and the nrDNA tree (with the exception of *B. nivea*; Fig. 2B) but were nested with the diploid species in the plastid tree (Fig. 2A). Cytonuclear conflicts in these polyploid species might indicate allopolyploid speciation, which is common in the formation of polyploidy (Morales-Briones *et al.*, 2018). The *B. crispa* complex and *B. alternifolia* each clustered as monophyletic groups in the ASTRAL species tree but were separated as polyphyletic groups in the plastid tree. *Buddleja crispa* is known to be involved in hybridization events with *B. alternifolia* (Liao *et al.*, 2021) and with *B. paniculata* (Liao *et al.*, 2015). Although most of the modern hybrids examined were F1s, the extensive contact and hybridization throughout the history of these species might have contributed to plastid capture in those lineages.

PhyloNet analysis verified the hybrid of *B. paniculata* and *B. crispa*, which was previously regarded mistakenly as a hybrid of *B. officinalis* and *B. crispa* (Liao *et al.*, 2015) owing to the misidentification of *B. paniculata*. The present study revealed ancestral introgression in the *B. crispa* complex, which might explain the cytonuclear discordance in this complex. Although gene flow from *B. alternifolia* to *B. crispa* was detected, we are unable to explain the polyphyletic nature of *B. alternifolia* in the plastid tree, particularly the unexpected position of three samples (RE120, RE121 and RE126) in Sichuan. This might be attributable to the fact that the species that originally caused the chloroplast capture has become extinct or remains unsampled (Li *et al.*, 2022) or it may have occurred long ago, with an ancestor of the *B. crispa* complex or *B. subcapitata* being involved in the hybridization that led to plastic transfer. Six polyploid species (*B. forrestii*, *B. nivea*, *B. albiflora*, *B. fallowiana*, *B. davidii* and *B. candida*) were shown to have undergone complex hybridization and genetic introgression (Fig. 4), which could explain the cytonuclear discordance in five of the species, although not that in *B. candida* (Fig. 2).

The origin of the hexaploid species *B. forrestii* might be a result of allopolyploidy, because one of its putative progenitors is tetraploid (*B. sessilifolia*). An allopolyploid origin of *B. forrestii* would explain why it grouped together with *B. sessilifolia* in the species tree (and is morphologically similar to *B. sessilifolia*; Fig. 1) but is widely separated from it in the plastid tree. Reticulate phylogenetic analysis indicated that hybridization and allopolyploidy might have played an important role in the diversification of the Asian *Buddleja*.

### *History of diversification in* Buddleja

Our results indicated that there were two stages of rapid diversification in the Asian *Buddleja* lineage (Fig. 5). The first stage occurred ~8–10 Mya, which might correspond to the last uplift in Hengduan Mountains and the intensification of the Asian monsoon (Favre *et al.*, 2015; Yang *et al.*, 2021). The second stage of rapid diversification might have occurred as a result of the Quaternary climate fluctuations (2.6 Mya; Clark *et al.*, 2009), which caused the radiation of many species in the Himalayas–Hengduan Mountains (Muellner-Riehl, 2019; Zhang *et al.*, 2021).

Extensive plateau uplift in the Miocene (5–15 Mya) intensified the summer monsoons, increasing the precipitation and erosion through river incision, leading to greater topographic relief (Herman *et al.*, 2013). Moreover, a remarkable increase in the intensity of silicate weathering at ~7–9 Mya, induced by the enhanced monsoons, caused massive CO_2_ consumption and fast global cooling (Yang *et al.*, 2021). This series of processes has not only accelerated the evolution of the biodiversity in the Himalayas–Hengduan Mountains (Ding *et al.*, 2020; Xu *et al.*, 2020), but also that of the monsoonal forests in South China (Kong *et al.*, 2022). The effect of climate modifications during the Quaternary ice age (0.1–2.6 Mya; Clark *et al.*, 2009) caused steep ecological gradients in mountainous areas (Wu *et al.*, 2022). At this time, rapid species radiation occurred in many mountainous areas, including the Himalayas, the Hengduan mountains, the Andes and the mountains of New Zealand (Hughes and Atchison, 2015). *Buddleja* Ser. Curviflorae Marq. comprises three species and has a disjunct distribution: *B. lindleyana* is found mainly on the Chinese mainland, whereas *B. curviflora* and *B. japonica* are found in Taiwan and Japan. The inferred time of divergence of *B. lindleyana* and the ancestor of *B. curviflora* and *B. japonica* was ~7.35 Mya (95 % HPD: 4.17–11.59 Mya). If this is correct, a Late Miocene landbridge across the East China Sea (~5.0–7.0 Mya; Kimura, 2003) would have allowed the common ancestor of *B. curviflora* and *B. japonica* to migrate from the Chinese mainland to Japan. Similar divergence times between other species with disjunct distributions in China and Japan have been found in *Euptelea* (Eupteleaceae; 6.39 Mya; Cao *et al.*, 2020) and *Deinanthe* (Hydrangeaceae; 7.1 Mya; Sakaguchi *et al.*, 2021). The inferred divergence time of *B. curviflora* from *B. japonica* was ~5.70 Mya (95 % HPD: 2.97–9.46 Mya), shortly after the formation of Taiwan Island (~6.5 Mya; Huang, 2017).

Our study suggests that a combination of tectonic activity, climate change, extensive hybridization and polyploidization might have contributed to the diversification of the Asian *Buddleja*.

## SUPPLEMENTARY DATA

Supplementary data are available online at https://academic.oup.com/aob and consist of the following. Table S1: sample and sequence information. Table S2: ploidy levels of Asian *Buddleja* species determined by flow cytometry and according to previous studies. Table S3: number of sample sequences and selection of subset samples in the PhyloNet analysis. Table S4: differences in morphological characters between *Buddleja asiatica* and *Buddleja subserrata*. Table S5: differences in morphological characters between *Buddleja caryopteridifolia* and sample GJ57. Table S6: differences in morphological characters between *Buddleja officinalis* and *Buddleja paniculata*. Fig. S1: the structure of the *Buddleja* chloroplast. Fig. S2: morphological comparison between *Buddleja asiatica* and *Buddleja subserrata*. Fig. S3: morphological comparison between the specimen GJ57 and *Buddleja caryopteridifolia* GJ31. Fig. S4: morphological comparison between *Buddleja officinalis* and *Buddleja paniculata*.

mcad022_suppl_Supplementary_Material

## Data Availability

The data that support the findings of this study can be found in online repositories. The names of the repository and accession number can be found below: https://db.cngb.org/search/project/CNP0003159/.
