# Supplementary material for: Deciphering complex reticulate evolution of Asian Buddleja (Scrophulariaceae): insights into the taxonomy and speciation of polyploid taxa in the Sino-Himalayan region
Source: Ann Bot. 2023 Feb 1;132(1):15–28. doi: 10.1093/aob/mcad022 (PMC10550280; doi:10.1093/aob/mcad022)
Supplement: mcad022_suppl_Supplementary_Material [file mcad022_suppl_supplementary_material.doc]

**Supplementary Tables**

**Table S1. Sample and sequence information**

| Sample ID | Species Name | Sampling Location | Voucher specimen | Data Accession | Genebank ID of Plastid | Genebank ID of nrDNA |
| --- | --- | --- | --- | --- | --- | --- |
| GJ1 | *Buddleja asiatica* | Yunnan, China | 1546858 | CNS0570461 | OP007375 | OP162553 |
| GJ2 | *Buddleja colvilei* | Nepal | 1546859 | CNS0570462 | OP007376 | OP162554 |
| GJ3 | *Buddleja colvilei* | Tibet, China | 1546860 | CNS0570463 | OP007377 | OP162555 |
| GJ4 | *Buddleja macrostachya* | Yunnan, China | 1546826 | CNS0570464 | OP007378 | OP162556 |
| GJ5 | *Buddleja lindleyana* | China, Yunnan, KBG (cultivated) | 1546828 | CNS0570465 | OP007379 | OP162557 |
| GJ6 | *Buddleja lindleyana* | Jiangxi, China | 1546829 | CNS0570466 | OP007380 | OP162558 |
| GJ7 | *Buddleja macrostachya* | Yunnan, China | 1546830 | CNS0570467 | OP007381 | OP162559 |
| GJ8 | *Buddleja curviflora* | Taiwan, China | 1546861 | CNS0570468 | OP007382 | OP162560 |
| GJ9 | *Buddleja sessilifolia* | Yunnan, China | 1546863 | CNS0570469 | OP007383 | OP162561 |
| GJ10 | *Buddleja sessilifolia* | Yunnan, China | 1546864 | CNS0570470 | OP007384 | OP162562 |
| GJ11 | *Buddleja sessilifolia* | Yunnan, China | 1546865 | CNS0570471 | OP007385 | OP162563 |
| GJ12 | *Buddleja salviifolia* | South Africa | J. Chau 252 (WTU) | CNS0570472 | OP007386 | OP162564 |
| GJ13 | *Buddleja americana* | China, Yunnan, KBG (cultivated) | 1546866 | CNS0570473 | OP007387 | OP162565 |
| GJ14 | *Buddleja aromatica* | Balivia | J. Chau 206 (WTU) | CNS0570474 | OP007388 | OP162566 |
| GJ15 | *Buddleja davidii* | Hubei, China | 1546867 | CNS0570475 | OP007389 | OP162567 |
| GJ16 | *Buddleja alternifolia* | Gansu, China | 1546870 | CNS0570476 | OP007390 | OP162568 |
| GJ18 | *Buddleja* sp. 2 | Yunnan, China | 022547 | CNS0570477 | OP007391 | OP162569 |
| GJ19 | *Buddleja candida* | Tibet, China | 1546871 | CNS0570478 | OP007392 | OP162570 |
| GJ20 | *Buddleja crispa* × *B. paniculata* | Yunnan, China | 1546874 | CNS0570479 | OP007393 | OP162571 |
| GJ21 | *Buddleja davidii* | Chongqing, China | 1546875 | CNS0570480 | OP007394 | OP162572 |
| GJ22 | *Buddleja delavayi* | Yunnan, China | 1546876 | CNS0570481 | OP007395 | OP162573 |
| GJ23 | *Buddleja fallowiana* | Yunnan, China | 1546877 | CNS0570482 | OP007396 | OP162574 |
| GJ24 | *Buddleja microstachya* | Yunnan, China | 1209697 | CNS0570483 | OP007397 | OP162575 |
| GJ26 | *Buddleja nivea* | Sichuan, China | 1546891 | CNS0570484 | OP007398 | OP162576 |
| GJ27 | *Buddleja paniculata* | Yunnan, China | 1546893 | CNS0570485 | OP007399 | OP162577 |
| GJ28 | *Buddleja paniculata* | Nepal | 1546895 | CNS0570486 | OP007400 | OP162578 |
| GJ29 | *Buddleja subcapitata* | Sichuan, China | 1209706 | CNS0570487 | OP007401 | OP162579 |
| GJ30 | *Buddleja yunnanensis* | Yunnan, China | 1546896 | CNS0570488 | OP007402 | OP162580 |
| GJ31 | *Buddleja caryopteridifolia* | Sichuan, China | 1543830 | CNS0570489 | OP007403 | OP162581 |
| GJ32 | *Buddleja* × *wardii* | Tibet, China | 1546898 | CNS0570490 | OP007404 | OP162582 |
| GJ33 | *Buddleja jinsixiaensis* | Shaanxi, China | RBZhu0156(WUK) | CNS0570491 | OP007405 | OP162583 |
| GJ34 | *Buddleja asiatica* | Tibet, China | 1546899 | CNS0570492 | OP007406 | OP162584 |
| GJ35 | *Buddleja crispa* | Tibet, China | 1546880 | CNS0570493 | OP007407 | OP162585 |
| GJ36 | *Buddleja tsetangensis* | Tibet, China | 1546882 | CNS0570494 | OP007408 | OP162586 |
| GJ37 | *Buddleja myriantha* | Yunnan, China | 1546884 | CNS0570495 | OP007409 | OP162587 |
| GJ38 | *Buddleja asiatica* | Laos | / | CNS0570496 | OP007410 | OP162588 |
| GJ40 | *Buddleja asiatica* | Tibet, China | 1546885 | CNS0570497 | OP007411 | OP162589 |
| GJ41 | *Buddleja asiatica* | Nepal | QTP-II08-T05-000367 | CNS0570498 | OP007412 | OP162590 |
| GJ42 | *Buddleja bhutanica* | Bhutan | E00850126 (K) | CNS0570499 | OP007413 | OP162591 |
| GJ43 | *Buddleja bhutanica* | Bhutan | E00850127 (K) | CNS0570500 | OP007414 | OP162592 |
| GJ44 | *Buddleja asiatica* | Guangxi, China | 1546886 | CNS0570501 | OP007415 | OP162593 |
| GJ45 | *Buddleja asiatica* | Yunnan, China | 1546887 | CNS0570502 | OP007416 | OP162594 |
| GJ46 | *Buddleja asiatica* | Yunnan, China | 1546889 | CNS0570503 | OP007417 | OP162595 |
| GJ47 | *Buddleja asiatica* | Yunnan, China | 1546841 | CNS0570504 | OP007418 | OP162596 |
| GJ49 | *Buddleja davidii* | Sichuan, China | 1546843 | CNS0570505 | OP007419 | OP162597 |
| GJ50 | *Buddleja asiatica* | Yunnan, China | 1546845 | CNS0570506 | OP007420 | OP162598 |
| GJ51 | *Buddleja forrestii* | Yunnan, China | 1546846 | CNS0570507 | OP007421 | OP162599 |
| GJ52 | *Buddleja brachystachya* | Yunnan, China | BSGLGSHL3336 | CNS0570508 | OP007422 | OP162600 |
| GJ53 | *Buddleja* sp.1 | Yunnan, China | BSGLGSLY3422 | CNS0570509 | OP007343 | OP162601 |
| GJ54 | *Buddleja davidii* | Shaanxi, China | 1546832 | CNS0570510 | OP007344 | OP162602 |
| GJ55 | *Buddleja albiflora* | Shaanxi, China | 1546833 | CNS0570511 | OP007345 | OP162603 |
| GJ56 | *Buddleja crispa* | Tibet, China | 1546835 | CNS0570512 | OP007346 | OP162604 |
| GJ57 | *Buddleja crispa* | Sichuan, China | 1543828 | CNS0570513 | OP007347 | OP162605 |
| GJ58 | *Buddleja asiatica* | Sichuan, China | 21SC20934 | CNS0570514 | OP007348 | OP162606 |
| GJ59 | *Buddleja fallowiana* | Yunnan, China | 1546836 | CNS0570515 | OP007349 | OP162607 |
| GJ60 | *Buddleja candida* | Tibet, China | 1546837 | CNS0570516 | OP007350 | OP162608 |
| GJ61 | *Buddleja officinalis* | Sichuan, China | 1546838 | CNS0570517 | OP007351 | OP162609 |
| GJ62 | *Buddleja davidii* | Sichuan, China | 1546823 | CNS0570518 | OP007352 | OP162610 |
| GJ63 | *Buddleja davidii* | Sichuan, China | 1546824 | CNS0570519 | OP007353 | OP162611 |
| GJ64 | *Buddleja nivea* | Sichuan, China | 1546825 | CNS0570520 | OP007354 | OP162612 |
| GJ65 | *Buddleja japonica* | Japan | 1591764 (PE) | CNS0570521 | OP007355 | OP162613 |
| GJ66 | *Buddleja japonica* | Japan | 1203616 (PE) | CNS0570522 | OP007356 | OP162614 |
| GJ67 | *Buddleja paniculata* | Nepal | 1546852 | CNS0570523 | OP007357 | OP162615 |
| GJ68 | *Buddleja lindleyana* | Sichuan, China | 1546853 | CNS0570524 | OP007358 | OP162616 |
| GJ69 | *Buddleja davidii* | Yunnan, China | 1546854 | CNS0570525 | OP007359 | OP162617 |
| GJ70 | *Buddleja paniculata* | Sichuan, China | 21CS20935 | CNS0570526 | OP007360 | OP162618 |
| GJ71 | *Buddleja paniculata* | Guizhou, China | 1546855 | CNS0570527 | OP007361 | OP162619 |
| GJ73 | *Buddleja microstachya* | Yunnan, China | 21CS20412 | CNS0570528 | OP007362 | OP162620 |
| GJ74 | *Buddleja* sp.1 × *Buddleja delavay*i | Yunnan, China | 1546856 | CNS0570529 | OP007363 | OP162621 |
| GJ75 | *Buddleja yunnanensis* | Yunnan, China | 1546857 | CNS0570530 | OP007364 | OP162622 |
| RE46 | *Buddleja alternifolia* | Gansu, China | / | SAMN13022400 | OP007365 | OP162623 |
| RE65 | *Buddleja alternifolia* | Gansu, China | / | SAMN13022382 | OP007366 | OP162624 |
| RE66 | *Buddleja alternifolia* | Gansu, China | / | SAMN13022383 | OP007367 | OP162625 |
| RE88 | *Buddleja tsetangensis* | Tibet, China | / | SAMN13022453 | OP007368 | OP162626 |
| RE110 | *Buddleja tsetangensis* | Tibet, China | / | SAMN13022432 | OP007369 | OP162627 |
| RE114 | *Buddleja tsetangensis* | Tibet, China | / | SAMN13022429 | OP007370 | OP162628 |
| RE120 | *Buddleja alternifolia* | Sichuan, China | / | SAMN13022423 | OP007371 | OP162629 |
| RE121 | *Buddleja alternifolia* | Sichuan, China | / | SAMN13022422 | OP007372 | OP162630 |
| RE123 | *Buddleja alternifolia* | Gansu, China | / | SAMN13022358 | OP007373 | OP162631 |
| RE126 | *Buddleja alternifolia* | Sichuan, China | / | SAMN13022418 | OP007374 | OP162632 |

Voucher specimens without parentheses are stored in the Herbarium, Kumming Institute of Botany, CAS (KUN). Sequences with data accession IDs starting with CNS are stored at CNGB (https://db.cngb.org/) and those starting with SAMN are stored at NCBI (https://www.ncbi.nlm.nih.gov/).

**Table S2**. Ploidy levels of Asian *Buddleja* species determined by FCM and according to previous studies

| **Species** | **Sample ID** | **Ploidy** | **Reference** | **Locality** |
| --- | --- | --- | --- | --- |
| *B. albiflora* |  | 6*x* | Moore (1947) | Hubei, China |
|  | GJ55 | 6*x* | This study | China, Shanxi, Baoji |
| *B. alternifolia* |  | 2*x* | Moore (1947) | Kansu, China |
|  |  | 2*x* | Moore (1947) | Qinghai, China |
|  | GJ16 | 2x | This study | Yinchuang, Gansu, China |
| *B. asiatica* |  | 2*x* | Moore (1947) | Malaya |
|  |  | 2*x* | Moore (1961) | S.E. India |
|  |  | 2*x* | Bir & Chatha (1983) | India |
|  |  | 2*x* | Gadella & Norman (1986) | India |
|  |  | 2*x* | Chatha & Bir (1987) | India |
|  |  | 2*x* | Sandhu & Mann (1988) | India |
|  |  | 2*x* | Chen et al. (2007) | Dali, Yunnan, China |
|  |  | 2*x* | Chen et al. (2007) | Chuxiong, Yunnan, China |
|  |  | 2*x* | Chen et al. (2007) | Wenshan, Yunnan, China |
|  | GJ1 | 2*x* | This study | Kunming, Yunnan, China |
|  | GJ34 | 2*x* | This study | Tibet, China |
| *B. brachystachya* |  | 2*x* | Chen et al. (2007) | Deqing, Yunnan, China |
|  | GJ52 | 4*x* | This study | Baoshan, Yunnan, China |
| *B. candida* |  | 4*x* | Moore (1960) | Kansu, China |
|  | GJ19 | 4*x* | This study | Tibet, China |
|  | GJ60 | 4*x* | This study | Nepal |
| *B. caryopteridifolia* |  | 2*x* | Janaki Ammal (1954) | W. China |
|  | GJ31 | 2*x* | This study | Shangri-La, Yunnan, China |
| *B. colvilei* |  | c. 16*x* | Moore (1947) | Himalayas |
|  |  | 8*x* | Janaki Ammal (1954) | N.E. India |
|  |  | 24*x* | Gadella (1980) | Nepal |
|  | GJ3 | 24*x* | This study | Tibet, China |
| *B. crispa* |  | 2*x* | Moore (1961) | N.E. India |
|  |  | 2*x* | Bedi et al. (1981) | Not given |
|  |  | 2*x* | Khatoon & Ali (1993) | Not given |
|  |  | 2*x* | Chen et al. (2007) | Deqing, Yunnan, China |
|  |  | 2*x* | Chen et al. (2007) | Kunming, Yunnan, China |
|  |  | 2*x* | Chen et al. (2007) | Yimen, Yunnan, China |
|  | GJ35 | 2*x* | This study | Tibet, China |
| *B. curviflora* |  | 2*x* | Chen et al. (2007) | Taiwan, China |
|  | GJ8 | 2*x* | This study | Taiwan, China |
| *B. davidii* |  | 4*x* | Moore (1947) | Central China |
|  |  | 4*x* | Chatha & Bir (1987) | S. India |
|  |  | 4*x* | Bir & Chatha (1983) | Not given |
|  |  | 4*x* | Chen et al. (2007) | Songming, Yunnan, China |
|  |  | 4*x* | Chen et al. (2007) | Zhaotong, Yunnan, China |
|  |  | 4*x* | Chen et al. (2007) | Suijiang, Yunnan, China |
|  | GJ69 | 4*x* | This study | Huize，Yunnan, China |
| *B. delavayi* |  | 6*x* | Moore （1960） | Yunnan, China |
|  | GJ22 | 6*x* | This study | Jianchuang, Yunnan, China |
| *B. fallowiana* |  | 4*x* | Moore (1947) | Yunnan, China |
|  |  | 4*x* | Chen et al. (2007) | Dali, Yunnan, China |
|  |  | 4*x* | Chen et al. (2007) | Zhongdian, Yunnan, China |
|  | GJ59 | 4*x* | This study | Lijiang, Yunnan, China |
| *B. forrestii* |  | 6*x* | Moore (1947) | Yunnan, China |
|  |  | 6*x* | Chen et al. (2007) | Dali, Yunnan, China |
|  |  | 6*x* | Chen et al. (2007) | Pianma, Yunnan, China |
|  |  | 6*x* | This study | Pianma, Yunnan, China |
| *B. japonica* |  | 2*x* | Moore (1947) | Japan |
|  |  | 2*x* | Chen et al. (2007) | Japan |
| *B. jinsixiaensis* | GJ33 | 2*x* | This study | Jinsixia, Shanxi, China |
| *B. lindleyana* |  | 2*x* | Moore (1947) | E. China |
|  |  | 2*x* | Chen et al. (2007) | Hubei, China |
|  | GJ5 | 2*x* | This study | KBG (cultivated), Yunnan, China |
| *B. lindleyana* | GJ68 | 2*x* | This study | Sichuan, China |
| *B. macrostachya* |  | 6*x* | Chen et al. (2007) | Ximeng, Yunnan, China |
|  |  | 6*x* | Chen et al. (2007) | Bingchuan, Yunnan, China |
|  |  | 6*x* | Chen et al. (2007) | Dehong, Yunnan, China |
|  |  | 6*x* | Chen et al. (2009) | Jianshui, Yunnan, China |
|  |  | 12*x* | Chen et al. (2007) | Simao, Yunnan, China |
|  |  | 12*x* | Chen et al. (2007) | Dali, Yunnan, China |
|  | GJ4 | 6*x* | This study | Pianma, Yunnan, China |
|  | GJ7 | 12*x* | This study | Wenshan, Yunnan, China |
| *B. microstachya* | GJ24 | 6*x* | This study | Yongde, Yunnan, China |
|  | GJ73 | 4*x* | This study | Yongde, Yunnan, China |
| *B. myriantha* |  | 2*x* | Janaki Ammal (1954) | Burma (Myanmar) |
|  |  | 4*x* | Chen et al. (2007) | Dali, Yunnan, China |
|  | GJ37 | 4*x* | This study | Gongshan, Yunnan, China |
| *B. nivea* |  | 6*x* | Moore (1947) | Sichuan, China |
|  |  | 12*x* | Moore （1960） | Yunnan, China |
|  | GJ64 | 12*x* | This study | Jinchuang, Sichuan, China |
| *B. officinalis* |  | 2*x* | Janaki Ammal (1954) | China |
|  |  | 2*x* | Chen et al. (2007) | Kunming, Yunnan, China |
|  |  | 2*x* | Chen et al. (2007) | Hekou, Yunnan, China |
|  | GJ61 | 2*x* | This study | Dujiangyan, Sichuan, China |
| *B. paniculata* |  | 2*x* | Janaki Ammal (1954) | Bhutan |
|  |  | 2*x* | Moore （1960） | Calcutta, India |
|  |  | 2*x* | Sandhu & Mann (1988) | India |
|  | GJ27 | 2*x* | This study | Kunming, Yunnan, China |
| *B. sessilifolia* | GJ10 | 4*x* | This study | Gongshan, Yunnan, China |
| *B. tsetangensis* | GJ36 | 2*x* | This study | Tibet, China |
| *B. yunnanensis* | GJ75 | 2*x* | Chen et al. (2007) | Simao, Yunnan, China |
|  | GJ57 | 2*x* | This study | Yunnan, China |
| *B.* sp. 1 | GJ53 | 6*x* | This study | Baoshan, Yunnan, China |
| *B.* *crispa* × *B. paniculata* | GJ20 | 2*x* | This study | Kunming, Yunnan, China |
| *B.* × wardii | GJ32 | 2*x* | This study | Tibet, China |
| *B.* sp. 1 × *delavayi* | GJ74 | 6*x* | This study | Baoshan, Yunnan, China |

**Table S3. Number of sample sequences and selection of subset samples in the PhyloNet analysis.**

| Samples ID | Species Names | Num_seqs  (Million) | Sequences  gaps | Sample selection |
| --- | --- | --- | --- | --- |
| GJ1 | *Buddleja asiatica* | 21.3 | 16.77% |  |
| GJ2 | *Buddleja colvilei* | 23.6 | 5.10% | √ |
| GJ3 | *Buddleja colvilei* | 24.8 | 6.25% |  |
| GJ4 | *Buddleja macrostachya* | 24.6 | 3.26% | √ |
| GJ5 | *Buddleja lindleyana* | 22.1 | 16.21% | √ |
| GJ6 | *Buddleja lindleyana* | 20.9 | 25.84% |  |
| GJ7 | *Buddleja macrostachya* | 21.5 | 5.00% |  |
| GJ8 | *Buddleja curviflora* | 19.0 | 18.90% |  |
| GJ9 | *Buddleja sessilifolia* | 21.5 | 7.52% | √ |
| GJ10 | *Buddleja sessilifolia* | 20.6 | 8.71% |  |
| GJ11 | *Buddleja sessilifolia* | 17.5 | 12.45% | √ |
| GJ12 | *Buddleja salviifolia* | 17.2 | 13.02% |  |
| GJ13 | *Buddleja americana* | 22.0 | 7.79% |  |
| GJ14 | *Buddleja aromatica* | 23.2 | 13.65% |  |
| GJ15 | *Buddleja davidii* | 21.6 | 24.75% |  |
| GJ16 | *Buddleja alternifolia* | 25.36 | 36.95% |  |
| GJ18 | *Buddleja* sp. 2 | 27.6 | 31.19% | √ |
| GJ19 | *Buddleja candida* | 18.1 | 9.58% | √ |
| GJ20 | *Buddleja crispa* × *B. paniculata* | 19.5 | 31.51% | √ |
| GJ21 | *Buddleja davidii* | 18.1 | 30.85% |  |
| GJ22 | *Buddleja delavayi* | 18.9 | 25.55% | √ |
| GJ23 | *Buddleja fallowiana* | 23.4 | 32.75% |  |
| GJ24 | *Buddleja microstachya* | 20.6 | 32.57% | √ |
| GJ26 | *Buddleja nivea* | 13.9 | 13.77% | √ |
| GJ27 | *Buddleja paniculata* | 19.4 | 12.89% | √ |
| GJ28 | *Buddleja paniculata* | 22.5 | 21.34% |  |
| GJ29 | *Buddleja subcapitata* | 25.8 | 10.25% | √ |
| GJ30 | *Buddleja yunnanensis* | 10.9 | 67.71% |  |
| GJ31 | *Buddleja caryopteridifolia* | 11.2 | 60.73% |  |
| GJ32 | *Buddleja* × *wardii* | 12.2 | 62.59% |  |
| GJ33 | *Buddleja jinsixiaensis* | 12.3 | 69.66% |  |
| GJ34 | *Buddleja asiatica* | 17.8 | 30.49% |  |
| GJ35 | *Buddleja crispa* | 16.3 | 13.63% | √ |
| GJ36 | *Buddleja tsetangensis* | 10.4 | 52.17% |  |
| GJ37 | *Buddleja myriantha* | 6.6 | 60.41% |  |
| GJ38 | *Buddleja asiatica* | 13.0 | 33.90% |  |
| GJ40 | *Buddleja asiatica* | 8.1 | 56.07% |  |
| GJ41 | *Buddleja asiatica* | 6.9 | 63.87% |  |
| GJ42 | *Buddleja bhutanica* | 23.5 | 66.95% |  |
| GJ43 | *Buddleja bhutanica* | 22.2 | 52.97% |  |
| GJ44 | *Buddleja asiatica* | 8.3 | 61.00% |  |
| GJ45 | *Buddleja asiatica* | 14.8 | 31.30% |  |
| GJ46 | *Buddleja asiatica* | 13.1 | 40.67% |  |
| GJ47 | *Buddleja asiatica* | 12.7 | 39.92% |  |
| GJ49 | *Buddleja davidii* | 13.4 | 25.91% |  |
| GJ50 | *Buddleja asiatica* | 15.1 | 32.68% |  |
| GJ51 | *Buddleja forrestii* | 15.8 | 18.13% | √ |
| GJ52 | *Buddleja brachystachya* | 14.9 | 21.58% | √ |
| GJ53 | *Buddleja* sp.1 | 17.5 | 13.52% | √ |
| GJ54 | *Buddleja davidii* | 14.5 | 14.47% |  |
| GJ55 | *Buddleja albiflora* | 16.8 | 9.95% | √ |
| GJ56 | *Buddleja crispa* | 15.9 | 14.30% | √ |
| GJ57 | *Buddleja crispa* | 21.1 | 12.08% | √ |
| GJ58 | *Buddleja asiatica* | 21.6 | 13.94% | √ |
| GJ59 | *Buddleja fallowiana* | 22.0 | 6.69% | √ |
| GJ60 | *Buddleja candida* | 19.5 | 9.68% |  |
| GJ61 | *Buddleja officinalis* | 20.4 | 12.06% | √ |
| GJ62 | *Buddleja davidii* | 17.0 | 19.32% |  |
| GJ63 | *Buddleja davidii* | 18.9 | 11.43% |  |
| GJ64 | *Buddleja nivea* | 19.1 | 9.66% | √ |
| GJ65 | *Buddleja japonica* | 26.7 | 20.80% |  |
| GJ66 | *Buddleja japonica* | 15.5 | 62.66% |  |
| GJ67 | *Buddleja paniculata* | 24.3 | 9.53% | √ |
| GJ68 | *Buddleja lindleyana* var. *sinuatodentata* | 25.6 | 9.58% |  |
| GJ69 | *Buddleja davidii* | 25.1 | 6.39% | √ |
| GJ70 | *Buddleja paniculata* | 9.8 | 42.98% |  |
| GJ71 | *Buddleja paniculata* | 28.7 | 6.53% | √ |
| GJ73 | Buddleja microstachya | 16.3 | 19.97% | √ |
| GJ74 | *Buddleja* sp.1 × *Buddleja delavay*i | 11.0 | 45.15% |  |
| GJ75 | Buddleja yunnanensis | 38.7 | 3.86% | √ |
| RE46 | *Buddleja alternifolia* | 20.0 | 15.76% |  |
| RE65 | *Buddleja alternifolia* | 20.0 | 9.02% |  |
| RE66 | *Buddleja alternifolia* | 20.0 | 9.12% | √ |
| RE88 | *Buddleja tsetangensis* | 20.0 | 7.48% | √ |
| RE110 | *Buddleja tsetangensis* | 20.0 | 6.67% |  |
| RE114 | *Buddleja tsetangensis* | 20.0 | 6.72% |  |
| RE120 | *Buddleja alternifolia* | 20.0 | 11.32% |  |
| RE121 | *Buddleja alternifolia* | 20.0 | 9.51% | √ |
| RE123 | *Buddleja alternifolia* | 20.0 | 9.64% |  |

**Table S4.** Differences in morphological characters between *Buddleja asiatica* and *B. subserrata*

| Leaf margin | Entire or subentire | Serrated lanceolate |
| --- | --- | --- |
| Inflorescence | Terminal and/or axillary; 1–3 or more racemose cymes together; clustered or less often interrupted | Spikes terminal, or base 1–3 cymes; interrupted |
| Corolla tube | 1.2–2 × 3–6 mm, 1.3–3 times as long as the calyx | 1–1.6 × 2.5–3.5 mm; 1.5–2.2 times as long as the calyx |
| Corolla lobe | 1–2 × 1–2.5 mm | 0.7–1.2 × 0.7–1.5 mm |
| Calyx | 1.5–4.5 mm long | 1–2.5 mm long |
| Leaf taste | Bitter | Sweet |

**Table S5.** Differences in morphological characters between *Buddleja caryopteridifolia* and sample GJ57

| Character | *B. caryopteridifolia* | GJ57 |
| --- | --- | --- |
| Leaf | 1–4 × 1–1.5 cm; margin dentate | 2.5–15 × 1–7 cm; margin crenate serrate |
| Inflorescence | 2–6 cm long | 4–10 cm long |
| Corolla tube | 6–9 mm, 1.3–3 times as long as the calyx | 9–12 mm; 2.5–4 times as long as the calyx |

**Table S6.** Differences in morphological characters between *Buddleja officinalis* and *B. paniculata*

| Character | *B. officinalis* | *B. paniculata* |
| --- | --- | --- |
| Corolla tube | (1.6–)2–2.2 × 9.5–13 mm | 1.2–1.6(–2) × 6–10mm |
| Corolla lobe | lilac, pinkish or pale purple | white or pale purple |
| Stamens | Inserted just above middle of corolla tube | Inserted just below corolla mouth |

**Supplementary Figures**


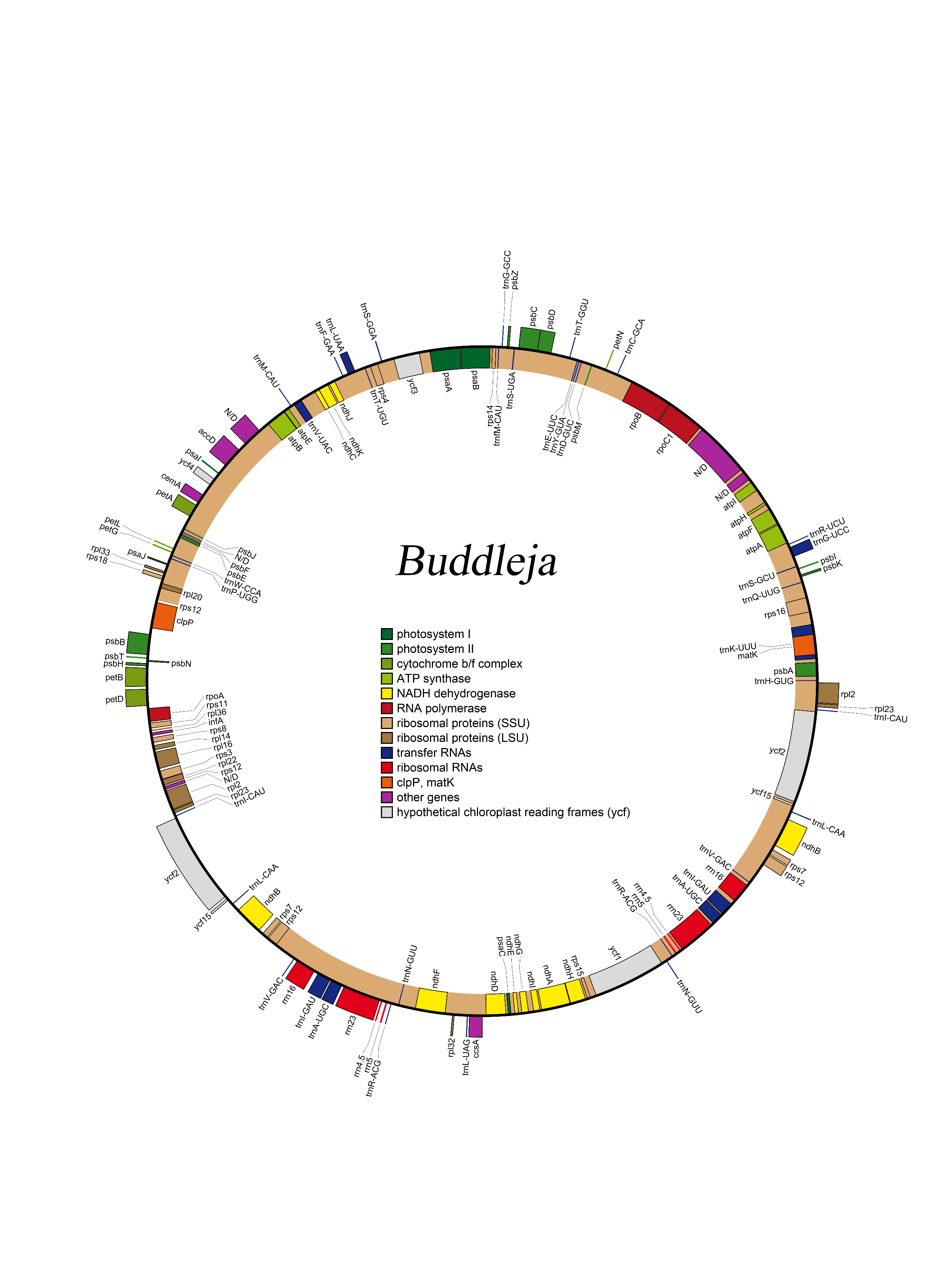


**Figure S1.** The structure of the *Buddleja* chloroplast

*

*

**Figure S2.** Morphological comparison between *Buddleja asiatica* collected in Yunnan, China (Voucher specimen 1546858 KUN!; A, C, E, G) and *B. subserrata* collected in Tibet, China. (Voucher specimen 1546885 KUN!; B, D, F, H). (A, B) Inflorescences, (C, D) leaves, (E, F) leaf margin (G, H) flower and corolla opened up.

*

*

**Figure S3.** Morphological comparison between the specimen GJ57 collected in Sichuan, China (1543828 KUN!; A, C) and *Buddleja caryopteridifolia* GJ31 collected in Yunnan, China (1543830 KUN!; B, D).





**Figure S4.** Morphological comparison between *Buddleja officinalis* collected in Sichuan, China (Voucher specimen 1546838 KUN!; A, C, E, G) and *B. paniculata* collected in Yunnan (Voucher specimen 1546893 KUN!; B, D, F, H). (A, B) Inflorescences, (C, D) leaves, (E, F) flower and corolla opened up, (G, H) front of corolla.

**References**

Bedi YS, Bir SS, Gill BS. 1981. Chromosome number reports LXXIII. *Taxon* 30: 843.

Bir SS, Chatha GS. 1983. SOCGI plant chromosome number reports – I. *Journal of Cytology and Genetics* 18: 56–58.

Chatha GS, Bir, SS. 1987. Population analysis of some woody species from Palni Hills, South India. *Journal of Cytology and Genetics* 22: 83–94.

Gadella TWJ. 1980. Cytology. In: Leeuwenberg AJM, ed. *Die Natürlichen Pﬂanzenfamilien*, 2nd edn., 28b. *I. Order Gentianales, Family Loganiaceae*. Berlin: Uuncker & Humblot, 202–210.

Gadella TWJ, Norman, EM. 1986. Chromosome number reports XCI. *Taxon* 35: 404–405.

Janaki Ammal EM. 1954. The cyto-geography of the genus *Buddleja* in Asia. *Science and Culture* 19: 578–581.

Khatoon S, Ali, SI. 1993. *Chromosome atlas of the angiosperms of Pakistan.* Karachi: Department of Botany, University of Karachi Press.

Moore R. 1947. Cytotaxonomic studies in the Loganiaceae. I. Chromosome numbers and phylogeny in the Loganiaceae. *American Journal of Botany* 34: 527–538.

Moore R. 1960. Cytotaxonomic notes on *Buddleja*. *American Journal of Botany* 47: 511–517.

Moore R. 1961. Polyploidy, phylogeny, and photoperiodism in Old World *Buddleja*. *Evolution* 15: 272–280.

Sandhu PS, Mann SK. 1988. SOCGI plant chromosome number reports – VII. *Journal of Cytology and Genetics* 23: 219– 228.
